# Multistage Combination Classifier Augmented Model for Protein Secondary Structure Prediction

**DOI:** 10.3389/fgene.2022.769828

**Published:** 2022-05-23

**Authors:** Xu Zhang, Yiwei Liu, Yaming Wang, Liang Zhang, Lin Feng, Bo Jin, Hongzhe Zhang

**Affiliations:** ^1^ College of Mechanical Engineering, Dalian University of Technology, Dalian, China; ^2^ School of Innovation and Entrepreneurship, Dalian University of Technology, Dalian, China; ^3^ The First Affiliated Hospital, Dalian Medical University, Dalian, China; ^4^ International Business School, Dongbei University of Finance and Economics, Dalian, China

**Keywords:** genetics, biology, protein secondary structure, deep learning, combination classifier, amino acid sequence

## Abstract

In the field of bioinformatics, understanding protein secondary structure is very important for exploring diseases and finding new treatments. Considering that the physical experiment-based protein secondary structure prediction methods are time-consuming and expensive, some pattern recognition and machine learning methods are proposed. However, most of the methods achieve quite similar performance, which seems to reach a model capacity bottleneck. As both model design and learning process can affect the model learning capacity, we pay attention to the latter part. To this end, a framework called Multistage Combination Classifier Augmented Model (MCCM) is proposed to solve the protein secondary structure prediction task. Specifically, first, a feature extraction module is introduced to extract features with different levels of learning difficulties. Second, multistage combination classifiers are proposed to learn decision boundaries for easy and hard samples, respectively, with the latter penalizing the loss value of the hard samples and finally improving the prediction performance of hard samples. Third, based on the Dirichlet distribution and information entropy measurement, a sample difficulty discrimination module is designed to assign samples with different learning difficulty levels to the aforementioned classifiers. The experimental results on the publicly available benchmark CB513 dataset show that our method outperforms most state-of-the-art models.

## Introduction

Gene controls the individual characters of biology through the guidance of protein synthesis to express its own genetic information. With the completion of the human genome project, scientists have never stopped studying the protein structure. Understanding protein secondary structure is very important for exploring diseases and finding new treatments (Huang et al., 2016; Li et al., 2021). Protein structure prediction is a very important research topic in the field of bioinformatics. Protein is the material basis of life activities, the basic organic matter of cells, and the main undertaker of life activities. Proteins can be folded into different structures or conformations, showing the feasibility of various biological processes in organisms. The protein structure determines its function, so the prediction of protein structure has great research value. In the field of bioinformatics, it is difficult to predict the spatial protein structure from the primary structure, so the prediction of the protein secondary structure has attracted much attention (Zhang, 2008; Källberg et al., 2012). Protein secondary structures refer to the local spatial structure of the polypeptide chain skeleton, not considering the conformation of the side chain and the spatial arrangement of the whole peptide chain. Besides, protein secondary structures are stabilized by hydrogen bonds on the backbone and are considered the linkages between primary sequences and tertiary structures (Myers and Oas, 2001). According to the distinct hydrogen bonding modes, generally, three types of secondary structures have been identified, namely helix (H), strand (E), and coil (C), where the helix and strand structures are most common in nature (Pauling et al., 1951). In the new classification calculated by the DSSP algorithm, the previous three states are extended to eight states, including α-helix (H), 3_10_ helix (G), π-helix (I), β-strand (E), β-bridge (B), β-turn (T), bend (S), and coil (C) (Kabsch and Sander, 1983), among which the α-helix and β-strand are the principal structure features (Lyu et al., 2021).

In the field of genetics and bioinformatics, protein secondary structure prediction is intended to predict the three-dimensional structure of a protein from its amino acid sequence (Drozdetskiy et al., 2015). The protein structure prediction is very important for understanding the relationship between protein structure and its function. Experimental protein structure determination methods include X-ray crystallography, nuclear magnetic resonance spectroscopy, and electron microscopy. However, all of these methods are very time-consuming and expensive and require expertise. What is more, at present, the growth rate of the protein sequence is much higher than the chemical or biological protein structure determination methods (Fang et al., 2020). Hence, it is very urgent to explore the protein secondary structure prediction methods. Although the three-dimensional structure of a protein cannot be accurately predicted directly from the amino acid sequence of the protein, we can predict the protein secondary structure to understand the three-dimensional structure of the protein. The protein secondary structure reserves part of the three-dimensional structure information and can help understand the three-dimensional morphology of the amino acid in the primary structure (Hanson et al., 2019).

Due to the high application value of the protein secondary structure prediction in many biological aspects, plenty of related algorithms based on deep learning methods have been proposed over the years (Li and Yu., 2016; Wang et al., 2016; Heffernan et al., 2017; Fang et al., 2018; Zhang et al., 2018; Uddin et al., 2020; Guo et al., 2021; Lyu et al., 2021; Drori et al., 2018). Current methods mainly utilize the convolutional and recurrent neural network to extract different protein features and then apply them to protein secondary structure prediction. For example, Li and Yu (2016) proposed an end-to-end deep network to predict the secondary structure of proteins from the integrated local and global context features, which leveraged convolutional neural networks with different kernel sizes to extract multiscale local contextual features and a bidirectional neural network consisting of the gated recurrent unit to capture global contextual features. Wang et al. (2016) presented Deep Convolutional Neural Fields (DeepCNF) for protein secondary structure prediction, which can model not only complex sequence-structure relationship by a deep hierarchical architecture but also interdependency between adjacent protein secondary labels, so it is much more powerful than traditional Convolutional Neural Fields. Lyu et al. (2021) presented a reductive deep learning model MLPRNN to predict either 3-state or 8-state protein secondary structures. Besides, Uddin et al. (2020) incorporated a self-attention mechanism within the Deep Inception-Inside-Inception network (Fang et al., 2018) to capture both the short- and long-range interactions among the amino acid residues. Guo et al. (2021). Integrated and developed multiple advanced deep learning architectures (DNSS2) to further improve secondary structure prediction. As described above, most researchers currently focus on exploring the complex deep learning models, and a few try to solve the protein secondary structure prediction task from the perspective of model learning or training methods, for example, “ELF: An Early-Exiting Framework for Long-Tailed Classification” (Duggal et al., 2020).

Real data usually follow a long-tailed distribution, most concentrated in only a few classes. On datasets following this distribution, neural networks usually cannot deal well with all classes (majority or rareness classes). If the model performs well on majority classes, it tends to perform poorly on the rareness classes and vice versa, resulting in poor performance. The protein secondary structure prediction task also shows a similar problem. For example, we visualize the CB6133-filtered and CB513 datasets (Zhou and Troyanskaya, 2014) and find an imbalance problem in the protein secondary structure labels distribution. For example, the number of labels α-helix (H), β-strand (E), and coil (C) is much greater than other labels. This imbalance problem has traditionally been solved by resampling the data (e.g., undersampling and oversampling) (Chawla et al., 2002; Minlong et al., 2019) or reshaping the loss function (e.g., loss reweighting and regularization) (Cao et al., 2019; Cui et al., 2019). However, by treating each example within a class equally, these methods fail to account for the important notion of example hardness. In other words, within each class, some examples are easier to classify than others (Duggal et al., 2020). Hence, “ELF: An Early-Exiting Framework for Long-Tailed Classification” is proposed to overcome the above-described limitations and address the challenge of data imbalance. ELF incorporates the notion of hardness into the learning process and can induce the neural network to increasingly focus on hard examples because they contribute more to the overall network loss. Hence, it frees up additional model capacity to distinguish difficult examples and can improve the classification performance of the model.

To our knowledge, few studies try to solve the protein secondary structure prediction task from the perspective of the model learning process. This study proposes a framework called Multistage Combination Classifier Augmented Model (MCCM) to solve that task and fill in the blanks. We first introduce a feature extraction module to extract features with different learning difficulty levels. Then, we design two classifiers that can learn the decision boundaries for easy and hard samples, respectively. Finally, we propose a sample learning difficulty discrimination module *via* exploring two strategies. Specifically, the first strategy is label-dependent, assuming the sample is hard if it is misclassified. However, the actual data is lack of labels. Hence, the second strategy utilizes Dirichlet distribution and information entropy measurement. The experimental results based on the first method and the benchmark CB513 dataset show that our proposed framework outperforms other state-of-the-art models by a large margin, indicating that if the multilevel samples discriminating module can be designed effectively, our framework can obtain state-of-the-art performance. Furthermore, the results based on the second method also show that our model outperforms most state-of-the-art models. In this work, we made the following key contributions:

• We are first to develop a Multistage Combination Classifier Augmented Framework for protein secondary structure prediction task. It consists of multilevel (easy or hard level in this study) features extraction, multistage combination classifiers, and multilevel samples discrimination module. The last module is realized based on label-dependent and label-independent methods, respectively.
• For our core multilevel samples discrimination module, a label-independent measurement standard to discriminate the easy and hard samples is first explored by our work based on Dirichlet distribution and information entropy theory. The Dirichlet distribution is designed to measure the model confidence based on subjective logic theory. The information entropy is designed to evaluate whether the Dirichlet distribution shows a highly confident distribution and, thus, capture the easy samples that tend to be classified accurately by the easy classifier.
• The results based on the label-independent method show that our model outperforms most state-of-the-art methods, indicating that the designed multilevel samples discrimination module herein is effective. The excellent result based on the label-dependent method means that our framework can obtain a state-of-the-art performance if the multilevel samples discriminating module is designed appropriately. Hence, our work not only offers a new idea to deal with the protein secondary structure prediction task but also leaves room for further research focusing on how to design a more effective multilevel samples discrimination module.

## Methods and Materials

### Benchmark Datasets

In the field of protein secondary structure prediction in genetics and bioinformatics, CB6133-filtered and CB513 datasets (Zhou and Troyanskaya, 2014) are two benchmark datasets widely used by the researchers (Li and Yu., 2016; Fang et al., 2018; Zhang et al., 2018; Guo et al., 2021; Lyu et al., 2021). The CB6133-filtered dataset is filtered to remove redundancy with the CB513 dataset (for testing performance on the CB513 dataset). In particular, the CB6133-filtered dataset is used to train the model, and the CB513 dataset is used to test the model. The training CB6133-filtered dataset is a large non-homologous sequence and structure containing 5,600 training sequences. The dataset is made by the PISCES Cull PDB server, a public server for screening protein sequence sets from the Protein Data Bank (PDB) according to sequence identification and structural quality standards (Wang and Dunbrick, 2003). The testing dataset CB513 was introduced by Cuff and Barton (Cuff and Barton, 1999, 2000), which contains 514 sequences. The two available benchmark datasets can be obtained by Zhou’s website.

### Input Features

Considering the difficulty of protein secondary structure prediction in genetics and bioinformatics, we use four types of features to characterize each residue in a protein sequence, including 21-dimensional amino acid residues, one-hot coding, and the sequence of 21-dimensional profile features, obtained from the PSI-BLAST (Altschul et al., 1997) log file and rescaled by a logistic function (Jones, 1999). Furthermore, the seven-dimensional physical property features (Jens et al., 2001) were previously used for the protein structure and property prediction by researchers (Heffernan et al., 2017) and obtained a good performance. The physical properties include steric parameters (graph-shape index), polarizability, normalized van der Waals volume (VDWV), hydrophobicity, isoelectric point, helix probability, and sheet probability. We also take them as one of the input features, and the features can be downloaded from Meiler’s study (Jens et al., 2001). Finally, the one-dimensional conservation score was obtained by applying the method (Quan et al., 2016):

$$R=\log20+\sum_{1}^{20}L_{i}\log L_{i}.$$ (1)

The residues are transformed according to the frequency distribution of amino acids in the corresponding column of homologous protein multiple sequence alignment. The score information in the profile features was calculated from this probability. Residue score in the *i*th column was calculated as follows (Altschul et al., 1997):

$$S_{i}=\left[\ln\left(\frac{L_{i}}{P_{i}}\right)\right]/\lambda_{u},$$ (2)

where $L_{i}$ is the predicted probability that a properly aligned homologous protein has amino acid *i* in that column and *P*_*i*_ is the background probability. $\lambda_{u}$ is 0.3176. $L_{i}$ is defined as

$$L_{i}=\exp\left(S_{i}\cdot \lambda_{u}\right)\cdot P_{i}.$$ (3)

### Model Design

The proposed model for protein secondary structure prediction in genetics and bioinformatics consists of a feature extracting module and a Multistage Combination Classifier Module. This section firstly introduces the two modules separately and then explains the overall architecture in detail.

### Multilevel Features Extraction

We use a multilevel features extraction module to extract easy (low level) and hard (high level) features. The easy feature is obtained through four linear layers and multiscale one-dimensional convolution layers. At first, we apply the four linear layers to the amino acid residues one-hot coding, sequence profile, physical property, and conservation score features, respectively. Further, we apply the concatenation function for the outputs, intended to obtain the feature representations with denser and more information. We define the concatenated outputs as

$$l=\;\left[l_{1}^{1},\ldots ,l_{T}^{1},l_{1}^{2},\ldots ,l_{T}^{2},l_{1}^{k},\ldots ,l_{T}^{k}\right],$$ (4)

where $l_{T}^{k}$ denotes the output of the linear layer k and T is the index of amino acid sequence. To model local dependencies of adjacent amino acids, we leverage multiscale one-dimensional convolution layers to extract local contexts (Li and Yu., 2016):

$$c_{i}=F\cdot l_{i:i+f-1}^{k}=\text{Relu}\left(w\cdot l_{i:i+f-1}^{k}+b\right),$$ (5)

where $F\in {\mathbb{R}}^{f\cdot m}$ is a convolutional kernel, f is kernel size, and m is the feature dimensionality of the concatenated outputs of the four linear layers l . w and b is the trainable parameters of the convolution layers. In this study, f of the three one-dimensional convolution layers is 5, 9, and 13, respectively. We define the concatenated outputs of the multiscale one-dimensional convolution layers as

$$c=\left[c_{1}^{1},\ldots ,c_{T}^{1},cl_{1}^{2},\ldots ,c_{T}^{2},cl_{1}^{k},\ldots ,c_{T}^{k}\right],$$ (6)

where $c_{T}^{k}$ denotes the output of the convolution layer k .

Then, based on the obtained easy feature c , the hard feature is further extracted by one Gate Recurrent Unit ( gru(⋅) ) and the attention mechanism. gru(⋅) is designed to capture the global contexts in the amino acid sequences. Defining the input of the GRU as ( $c_{t}^{k},\;h_{t-1}$ ), the mechanism of a GRU can be presented as follows:

$$r_{t}=sigm\left(W_{cr}\cdot c_{t}+W_{hr}\cdot h_{t-1}+b_{r}\right),$$ (7)

$$u_{t}=sigm\left(W_{cu}\cdot c_{t}+W_{hu}\cdot h_{t-1}+b_{u}\right),$$ (8)

$${\tilde{h}}_{t}=tanh\left(W_{c\tilde{h}}\cdot c_{t}+W_{h\tilde{h}}\cdot \left(r_{t}⊙h_{t-1}+b_{\tilde{h}}\right)\right),$$ (9)

$$h_{t}=u_{t}⊙h_{t-1}+\left(1-u_{t}\right)⊙{\tilde{h}}_{t},$$ (10)

where $r_{t}$ is the activation of the reset gate; $u_{t}$ is the activation of the update gate; ${\tilde{h}}_{t}$ is the internal memory cell; $h_{t}$ is the GRU output; $W_{cr}$ , $W_{cr}$ , $W_{cu}$ , $W_{hu}$ , $W_{c\tilde{h}}$ , and $W_{h\tilde{h}}$ are weight matrices; and $b_{r}$ , $b_{u}$ , and $b_{\tilde{h}}$ are bias terms. Besides, ⊙ , sigm , and tanh denote element-wise multiplication, sigmoid, and hyperbolic functions, respectively. Further, the sequential attention mechanism (SAM) has been widely used in the LSTM-based solutions for sequential learning problems (Feng et al., 2019). In this study, considering the global contexts $h_{t}$ of different amino acid sequence steps could contribute differently to the representation of the whole amino acid sequences. We use the attention mechanism to compress the hidden representations of global contexts $h_{t}$ at different sequence steps into an overall representation with adaptive weights:

$${\tilde{\alpha }}_{t}=s_{a}^{T}\tanh\left(W_{a}\cdot h_{t}+b_{a}\right),$$ (11)

$$\alpha_{t}=\frac{\exp^{{\tilde{\alpha }}_{t}}}{\sum_{t=1}^{T}\exp^{{\tilde{\alpha }}_{t}}},$$ (12)

$$\alpha_{h}=\sum_{t=1}^{T}\alpha_{t}h_{t},$$ (13)

where $W_{a}$ , $s_{a}$ , and $b_{a}$ are trainable parameters and $\alpha_{h}$ denotes the important contexts information, aggregating from the global contexts $h_{t}$ . Although $\alpha_{h}$ aggregated most part of the important contexts’ information, it also may lead to losing part of important information more or less. Hence, we apply the concatenation function to the original global contexts $h_{t}$ and the aggregated contexts information $\alpha_{h}$ . At last, the obtained local contexts, global contexts, and aggregated global contexts through SAM are concatenated together as the hard features:

$$v=\left[c,h,\alpha_{h}\right].$$ (14)

Finally, the easy feature c is sent to the easy classifier, and the hard feature v is sent to the hard classifier.

#### Multistage Combination Classifier Module

Predicting protein secondary structure in genetics and bioinformatics is a challenging task that we try to solve from the perspective of the model learning method. On the one hand, the existing research results point out that, within different classes of all samples (the classes are either majority or minority), some examples are easier than the others (Duggal et al., 2020). On the other hand, different people may be suitable for different work. Similarly, the different classifiers may be suitable for classifying different samples. Following the theory and intuition, we design two classifier branches in the model to deal with samples with different difficulty levels. The first classifier branch comprises a simple linear layer, which aims to deal with the simple samples (easy to classify). The second classifier branch comprises a multi-layer perceptron ( MLP(⋅) ), which is more complex than the first classifier and aims to deal with the hard samples (hard to classify). The first classifier branch can correctly classify some easy samples and serve as a filter to filter them out, avoiding being sent to the second complex classifier. We regard the remaining samples, classified by the first classifier incorrectly, as hard samples and further send them to the second complex classifier. After the computation of each classifier, we calculate the cross-entropy loss between the predicted outputs and ground truth labels:

$${ℒ}_{\text{C}}=\frac{1}{B}\sum_{i}^{B}-\sum_{j=1}^{Z}y_{ij}\left(\log\left(p_{ij}\right)\right),$$ (15)

where B is the number of batch samples and Z is the number of target labels. We can further obtain the cross-entropy loss computed based on the easy and hard classifier and describe them as ${ℒ}_{\text{C}\_\text{easy}}$ and ${ℒ}_{\text{C}\_\text{hard}}$ , respectively. After the computation of the first simple classifier, we can obtain the loss value of all samples. Further, we can obtain the loss value of the hard samples after the computation of the second hard classifier. Hence, the loss value of the harder samples is increased in general, and the model is induced to pay more attention to harder samples and improve the classification performance. The final loss function is the sum of the cross-entropy loss of the easy and hard versions:

$${ℒ}_{\text{C}\_\text{all}}={ℒ}_{\text{C}\_\text{easy}}+{ℒ}_{\text{C}\_\text{hard}}.$$ (16)

#### Sample Difficulty Discrimination Module

**FIGURE 1:**
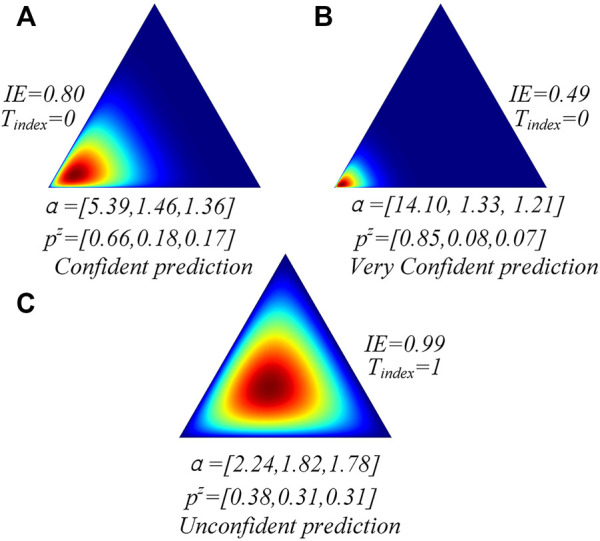
Prediction of the Dirichlet distribution of the three amino acid samples’ analysis.

We have designed the easy and hard classifiers to deal with different samples. However, we need to further design a measurement standard to discriminate between the easy and hard samples among all samples. For the model, a label is an ideal tool to realize our purpose. If samples are classified accurately according to their labels, we regard them as easy samples and the others as hard samples sent to the hard classifier. In this way, the different classifiers can be assigned suitable samples, and our model can be trained well. However, the actual data is lack of labels. Hence, we just can design the measurement standard to get close to the ideal effect in all possible ways. In this study, we design a measurement standard based on subjective logic (SL) and Dirichlet distribution with Z parameters $\alpha =\left[\alpha_{1},\alpha_{2},\ldots ,\alpha_{Z}\right]$ , and α are called subjective opinions (Dempster, 2008; Josang, 2016). If the model has a highly motivated subjective opinion on one class of one test sample, it means that the model is confident to classify it accurately after being trained on the training data. For example, as shown in Figure 1, the easy classifier classifies the amino acid into three states (H, E, and C). IE denotes the information entropy, $T_{index}$ denotes the index of True label, and $p^{z}$ denotes the expectation of the Dirichlet distribution. We will further discuss them in the following part. Based on the subjective logic theory, we know that if the predicted α of certain amino acids are [14.10, 1.33, and 1.21] (each α corresponds to one state), the easy classifier is very confident to classify the amino accurately. Hence, following a Dirichlet distribution, the subjective multinomial opinion will yield a sharp distribution on one corner of the simplex (Figures 1A, B). However, if the predicted α are [2.24, 1.82, and 1.78], as shown in Figure 1C, the model is not confident to classify it accurately, and it should be sent to a hard classifier. In this condition, the multinomial opinion will yield a central distribution (Figure 1B). The Dirichlet distribution is a probability density function (pdf) for possible values of the probability mass function (pmf) p and can be expressed by *Z* parameters α :

$$Dir\left(p|\alpha \right)=\{\begin{array}{c} \frac{1}{B\left(\alpha \right)}\overset{Z}{\underset{i=1}{\prod }}p_{i}^{\alpha_{i-1}},\;\\ \begin{array}{cc} 0 & otherwise \end{array} \end{array},$$ 17)

where p is the probability mass function and $\alpha =\left[\alpha_{1},\ldots ,\alpha_{Z}\right]$ are the parameters of Dirichlet distribution. Z denotes the label category. B(α) is a polynomial beta function in Z dimension [36]. Based on SL, the expectation of Dirichlet distribution based on neural network evidence theory can be computed as follows:

$$p^{z}=\alpha_{z}/S,$$ (18)

$$\text{S}=\sum_{z=1}^{Z}\alpha_{z},$$ (19)

$$\alpha_{z}=e_{z}+1,$$ (20)

$$e_{z}=\zeta \left({\hat{y}}^{s}\right),$$ (21)

where $\alpha_{z}$ are Dirichlet parameters, ${\hat{y}}^{s}$ is the output vector before being sent to the *softmax* layer, and ζ(⋅) denotes an activation layer (e.g., ReLU). $e_{z}$ is the amount of evidence and S is the Dirichlet strength. By minimizing the mean square error loss based on the Dirichlet parameters, the Dirichlet distribution can be optimized according to the loss function as

$$L^{dir}=\sum_{b=1}^{B}{\left(y_{b}-p_{b}\right)}^{2}+\frac{p_{b}\left(1-p_{b}\right)}{\left(S_{b}+1\right)},$$ (22)

where B is the batch size of the samples, $y_{b}$ is the real label of a single sample, $p_{b}$ is the Dirichlet distribution expectation of a single sample, and $S_{b}$ is the Dirichlet strength of a single sample.

Finally, we use the information entropy (IE) to know whether the easy classifier has a highly motivated subjective opinion on the samples. Given the predicted Dirichlet distribution parameters $\left[\alpha_{1},\alpha_{2},\ldots ,\alpha_{Z}\right]$ , we can compute $p^{z}$ . Further, we can compute the information entropy of $p^{z}$ , which is defined as

$$H\left(p^{z}\right)=-\sum p\left(p^{z}\right)log_{2}\left(p^{z}\right).$$ (23)

As shown in Figure 1, if the easy classifier is very confident to classify the sample accurately, its information entropy tends to be lower than other conditions. We can also find that the classifier with low information entropy shows a highly motivated subjective opinion on the current samples and classifies them accurately (Figures 1A,B). However, the classifier with high information entropy shows a uniform subjective opinion on the current sample labels and classifies it incorrectly. Hence, the information entropy can be used to help the model discriminate between the easy and hard samples. We define the discriminating process as

$$samples_{cur}\;\;=\{\begin{array}{cc} samples_{easy}, & if\;argmax\left(p_{b}\right)=y_{b}\;and\;H\left(p^{z}\right)<H{\left(p^{z}\right)}_{per}\\ samples_{hard}, & otherwise \end{array},$$ (24)

where $H{\left(p^{z}\right)}_{per}$ is *per* th percentile of $H\left(p^{z}\right)$ , which is a threshold to distinguish the samples into hard or easy samples. In particular, in the training process, if the samples are classified correctly and their information entropy is lower than the threshold, they will not be sent to the hard classifier. Otherwise, the samples will be sent to the hard classifier again. In the test process, there is no need to know whether the samples are classified correctly, and the easy or hard samples are only divided based on the information entropy. The samples with the high information entropy will be sent to the hard classifier for the final prediction result.

Hence, the final loss function of our model can be obtained by uniting Eqs. 16, 22:

$${ℒ}_{\text{C}\_\text{final}}={ℒ}_{\text{C}\_\text{easy}}+\beta \cdot L_{easy}^{dir}+{ℒ}_{\text{C}\_\text{hard}}+\beta \cdot L_{hard}^{dir}.$$ (25)

According to Eq. 22, $L_{easy}^{dir}$ and $L_{hard}^{dir}$ are calculated based on the output of the easy and hard classifiers, respectively.

**FIGURE 2:**
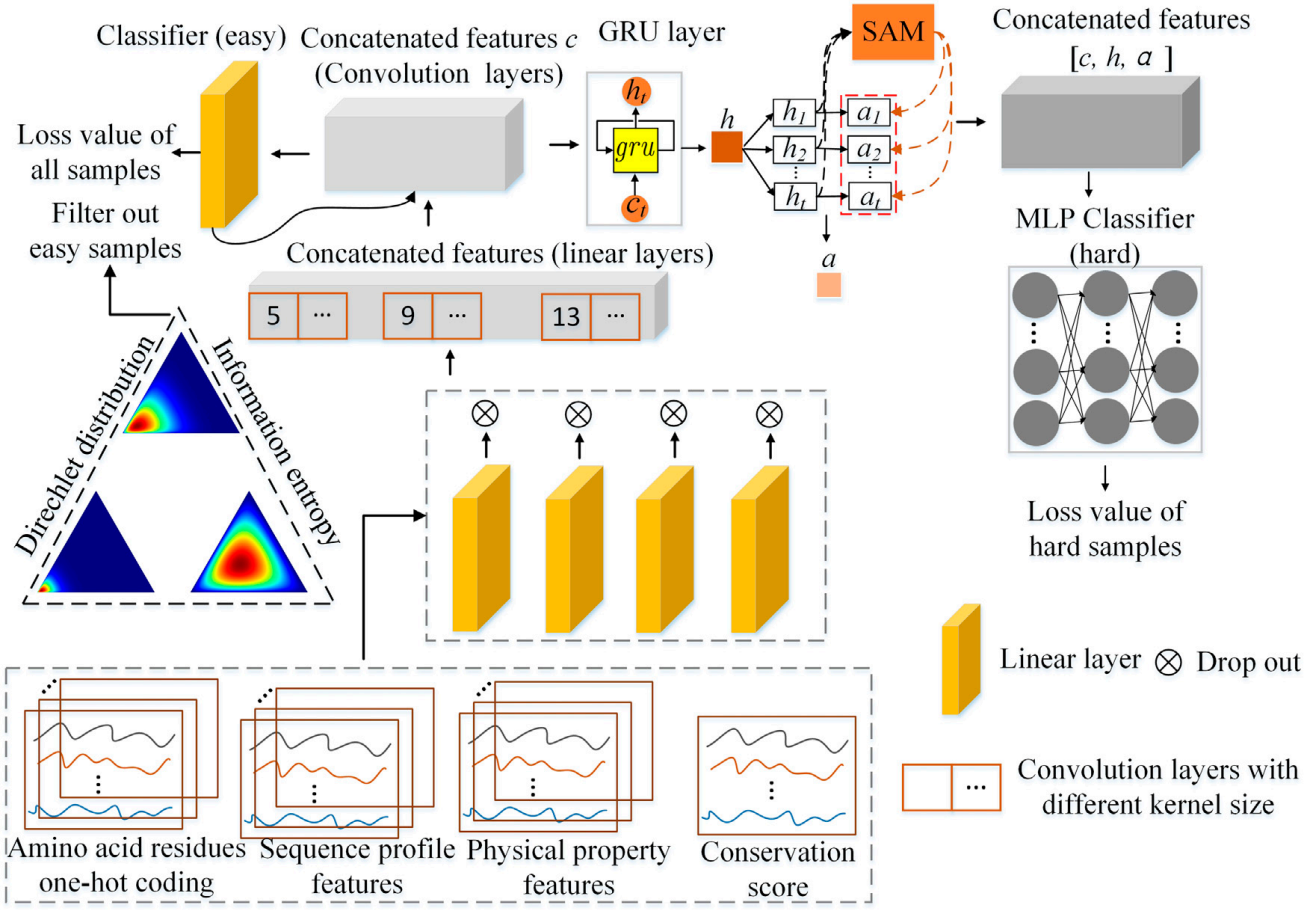
The overall architecture of the Multistage Combination Classifier Augmented Model (MCCM) for protein secondary structure prediction in genetics and bioinformatics.

The architecture of the Multistage Combination Classifier Augmented Model (MCCM) is shown in Figure 2. After preprocessing the dataset, 50-dimensional features are obtained and taken as the input features, including the 21-dimensional amino acid residues one-hot coding, 21-dimensional sequence profile, 7-dimensional physical property, and 1-dimensional conservation score. The features are first preprocessed into easy ones through four linear layers and multiscale one-dimensional convolution layers. Based on the Dirichlet distribution and information entropy, the samples are divided into easy and hard ones by an easy classifier (a simple linear layer). Then, the easy feature is further preprocessed into a hard one through gru(⋅) and the attention mechanism SAM. Finally, the hard samples are sent into a hard classifier ( MLP(⋅) ).

### Implementation Details

The hidden sizes of the four linear layers used for the 21-dimensional amino acid residues one-hot coding, 21-dimensional sequence profile, 7-dimensional physical property, and 1-dimensional conservation score features are 64, 128, 32, and 16, respectively. The hidden size of the multiscale one-dimensional convolution layers is 64 and the corresponding kernel sizes are 5, 9, and 13, respectively. The GRU layer is bidirectional and the hidden size is 256. The hidden size of the linear layer used in the attention mechanism is 256. The hidden sizes of the first two layers used in MLP are 512 and 1,024. The models are optimized by Adam optimizer, and the learning rates are set to 0.0005. During training, the dropout function can randomly zero some of the elements of the input tensor with probability τ using samples from a Bernoulli distribution. Herein, the dropout function is used in four linear layers and the MLP layers and τ is set as 0.5. The percentile of $H\left(p^{z}\right)$ used in this study is 15, 30, and 35. The β used in Eq. 22 is 1. All results have been produced based on the same hardware environment: Intel (R) Core (TM) CPU I7-10700 @ 2.90 GHz 16 cores. Finally, we define the proposed label-dependent model (only used to explore the theoretical best performance) equipped with both ${ℒ}_{\text{C}\_\text{easy}}$ and ${ℒ}_{\text{C}\_\text{hard}}$ as MCCM. The proposed label-dependent model equipped with only ${ℒ}_{\text{C}\_\text{easy}}$ is defined as MCCM_easy_, which means there is no backpropagation operation through the loss function ${ℒ}_{\text{C}\_\text{hard}}$ . The proposed label-independent model (use the evidence and information entropy theory to divide the samples into easy and hard ones) is MCCM_dir_.

### Performance Evaluation

In the field of protein secondary structure prediction in genetics and bioinformatics, the Q score measurement formulated as Eq. 6 has been widely used to evaluate the performance of the proposed models. It measures the percentage of residues for which the predicted secondary structures are correct (Wang et al., 2016):

$$Q_{k}=100\%\times \frac{\sum_{i=1}^{k}N_{correct}\left(i\right)}{N},$$ (26)

where k indicates the number of classes, for example, $Q_{3}$ score ( k = 3) or $Q_{8}$ score ( k = 8). $Q_{8}$ classes include α-helix (H), 3_10_ helix (G), π-helix (I), β-strand (E), β-bridge (B), β-turn (T), bend (S), and coil (C). $Q_{8}$ is transformed to $Q_{3}$ by treating the label (B, E) as E, (G, I, H) as H, and (S, T, C) as C.

## Results and Discussion

### Experimental Results of Evaluating Indicators

The evaluation results of the proposed model based on the public CB513 test dataset are shown in Table 1. MCCM_dir_ means using the Dirichlet distribution and information entropy to divide the samples into easy and hard ones. In MCCM_easy_ and MCCM, we use label information to divide the samples, an ideal measurement method that can help us explore the theoretical best performance. Besides, MCCM_easy_ means there is no backpropagation operation through the loss function ${ℒ}_{\text{C}\_\text{hard}}$ and the model will not be induced to pay more attention to hard samples. The results of the benchmark methods on CB513 datasets are obtained from (Shapovalov et al., 2020), except for the DNSS2, which is obtained from Guo et al. (2021). We can find that the Q3 and Q8 accuracy of the MCCM_dir_ is better than most of the benchmark methods, denoting that the designed method is based on Dirichlet distribution and information entropy to distinguish the hard or easy samples is effective. Besides, note that, due to our computer resource constraints, there are only two designed classifiers and corresponding feature extractors in our framework, which limits the performance of our model. Moreover, compared with MCCM_easy_, we can find that MCCM outperforms state-of-the-art models by a large margin in both Q3 and Q8 accuracies, which means that the model is induced to pay more attention to hard samples and improves the classification performance of the model overall through the backpropagation operation of both ${ℒ}_{\text{C}\_\text{easy}}$ and ${ℒ}_{\text{C}\_\text{hard}}$ . It shows that it is reasonable to use different classifiers to classify samples with different difficulty levels, thus increasing the loss values of hard samples, inducing the model to pay more attention to hard samples. However, the label information is lacking in actual data, so the excellent performance of MCCM only denotes that if the multilevel samples discriminating module (divide samples into hard and easy ones) is designed very effectively, our method can obtain the state-of-the-art performance.

**TABLE 1:** Q3 and Q8 accuracy of different algorithms on the public CB513 dataset.

Algorithms	Q3	Q8
DeepCNF (2016)	81.80	69.1
DCRNN (2018)	-	69.70
eCRRNN (2018)	81.20	70.2
DNSS2 (2021)	82.56	73.36
BLSTM (2015)	-	67.40
GSN (2014)	-	66.40
SSpro, free (2014)	78.50	63.50
JPRED4 (2015)	81.70	-
SecNet (2020)	84.30	72.30
MCCM_dir_	82.12	69.79
MCCM_easy_	86.94	71.78
MCCM	**96.31**	**83.74**

The bold values denote the best values of performance metrics.

### Ablation Study

This section gives a more comprehensive analysis regarding the effectiveness of the proposed framework. The different levels of classifiers equipped with the multilevel samples discriminating module can improve the performance of the prediction task. The compared variants are as follows:

MCCM_c1_: the variant is the front part of our proposed model (without the multilevel classifiers and sample difficulty discrimination module), which only uses the concatenated features *c* and easy classifier (see Figure 2) to deal with the prediction task. This model only uses the convolution layer to extract the features and then conducts the classification task based on the low-level features.

MCCM_c2_: the variant is the part of our proposed model (without the multilevel classifiers and sample difficulty discrimination module) that uses the concatenated features [*c,h,a*] and hard classifier (see Figure 2) to deal with the prediction task. This model uses not only the convolution layer but also gru(⋅) and the attention mechanism to extract the features. Then, it conducts the classification task based on both the low- and high-level features.

MCCM_conf_: the variant is designed based on the ELF (Duggal et al., 2020), which uses the classifier confidence to distinguish the samples into easy and hard ones. Particularly, in the training process, if the samples are classified correctly and their classifier confidence is lower than the threshold (0.9 used in ELF and this study), they will not be sent to the hard classifier. Otherwise, the samples will be sent to the hard classifier again. In the test process, the samples with low classifier confidence will be sent to the hard classifier for the final prediction result.

The experiment results are shown in Table 2. The performance of MCCM_c1_ is the worst, and the performance of MCCM_c2_ is better than it, which means that the addition of the high-level features extractor GRU and attention mechanism is effective. The performance of MCCM_dir_ is better than MCCM_c2_, which means that our proposed framework is effective. The designed multilevel classifiers and sample difficulty discrimination module can help the model pay more attention to the hard samples and improve the model performance. Note that, if we can increase the depth of our network and use more classifier branches, the model performance will be better. Moreover, the performance of MCCM_dir_ is better than that of MCCM_conf_, which means that our designed sample difficulty discrimination module is better than that proposed in ELF (Duggal et al., 2020). The Dirichlet distribution united with the information entropy can divide the samples into hard and easy ones, which is better than using the simple classifier confidience.

**TABLE 2:** Q3 and Q8 accuracy of variant models on the public CB513 dataset.

Algorithms	Q3	Q8
MCCM_c1_	79.93	66.30
MCCM_c2_	81.45	68.92
MCCM_conf_	81.00	66.42
MCCM_dir_	**82.12**	**69.79**

The bold values denote the best values of performance metrics.

### Analysis of the Training Process

**FIGURE 3:**
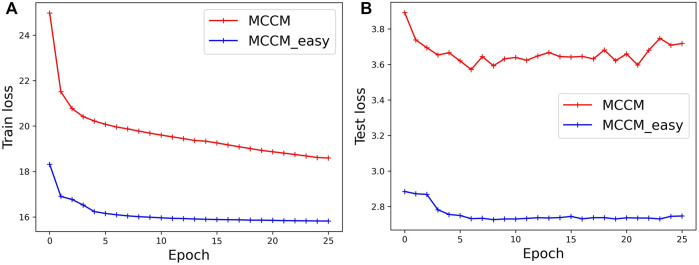
The loss value computed on the training and test datasets.

The training loss computed on the CB6133-filtered dataset and the test loss computed on the CB513 dataset are shown in Figure 3. Label-dependent MCCM and label-independent MCCM_dir_ are all optimized by the loss functions ${ℒ}_{\text{C}\_\text{easy}}$ and ${ℒ}_{\text{C}\_\text{hard}}$ , but MCCM_easy_ is only optimized by the loss function ${ℒ}_{\text{C}\_\text{easy}}$ . Hence, the loss value of the MCCM is always greater than that of MCCM_easy_. ${ℒ}_{\text{C}\_\text{hard}}$ is computed based on the hard samples and can induce the model to pay more attention to the hard samples (samples tend to be classified incorrectly in both majority or rareness classes).

### Analysis of the Prediction of Q8 States and Confusion Matrix

In the field of protein secondary structure prediction in genetics and bioinformatics, the predictive precision for each class of Q8 would provide more useful information, and we compute the prediction accuracy of each label in the Q8 states based on the CB513 dataset. At the same time, we compute the confusion matrix to further explore the model performance. Table 3 shows the prediction accuracy (MCCM_dir_, MCCM_easy_, and MCCM) of each label in the Q8 states. Tables 4, 5 show the Q3 and Q8 prediction confusion matrix, respectively. Associating the three tables, we can find that although MCCM_easy_ is only optimized by the loss function ${ℒ}_{\text{C}\_\text{easy}}$ , it also outperforms MCCM_dir_. The main reason for the better performance is that MCCM_easy_ uses the label information to divide the samples into hard and easy ones, denoting that the method to distinguish the samples is very important.

**TABLE 3:** Prediction accuracy of each label in the Q8 states based on the CB513 dataset.

Label	Types	Frequency	MCCM_dir_	MCCM_easy_	MCCM
H	α-Helix	30.86	91.97	89.91	**96.30**
E	β-Strand	21.25	83.67	80.08	**92.30**
C	Coil	21.14	63.73	63.92	**88.45**
T	β-Turn	11.81	53.96	**88.46**	74.37
S	Bend	9.81	26.35	23.68	**51.91**
G	3_10_ Helix	3.69	30.62	18.3	**46.87**
B	β-Bridge	1.39	4.57	3.47	**6.94**
I	π-Helix	0.04	0.00	**3.33**	**0.00**

The bold values denote the best values of performance metrics.

**TABLE 4:** $Q_{3}$ confusion matrix, of 84,765 test labels (MCCM_dir_, MCCM_easy_, and MCCM).

Accuracy (MCCM_dir_)		Pred freq.	True label		
82.12			C	E	H
True freq.		100%	42.76	22.65	34.59
Predicted label	C	44.53	**35.15**	3.76	3.85
	E	21.07	5.27	**16.90**	0.48
	H	34.40	4.11	0.41	**30.07**

Accuracy (MCCM_easy_)		Pred freq.	True label		
86.94			C	E	H
True freq.		100%	42.76	22.65	34.59
Predicted label	C	36.75	**36.1**	4.87	1.79
	E	27.66	0.38	**19.65**	2.61
	H	35.59	0.26	3.14	**31.19**

Accuracy (MCCM)		Pred freq.	True label		
96.31			C	E	H
True freq.		100%	42.76	22.65	34.59
Predicted label	C	42.41	**41.75**	0.29	0.72
	E	22.59	0.47	**21.22**	0.95
	H	35.00	0.19	1.07	**33.33**

The bold values denote the best values of performance metrics.

**TABLE 5:** $Q_{8}$ confusion matrix of 84,765 test labels (MCCM_dir_, MCCM_easy_, and MCCM).

Accuracy (MCCM_dir_)		Pred freq.	True label							
69.79			C	B	E	G	I	H	S	T
True freq.		100%	21.14	1.39	21.25	3.69	0.04	30.86	9.81	11.81
Predicted label	C	23.33	**13.47**	0.04	3.58	0.27	0.00	1.04	1.28	1.46
	B	0.14	0.69	**0.06**	0.33	0.02	0.00	0.11	0.09	0.09
	E	23.81	2.29	0.02	**17.78**	0.07	0.00	0.42	0.31	0.37
	G	2.57	0.65	0.00	0.21	**1.13**	0.00	0.91	0.12	0.68
	I	0.00	0.00	0.00	0.00	0.00	**0.00**	0.03	0.00	0.00
	H	33.43	0.86	0.01	0.29	0.4	0.00	**28.38**	0.1	0.82
	S	5.12	3.48	0.01	1.07	0.18	0.00	0.69	**2.58**	1.79
	T	11.6	1.9	0.	0.55	0.5	0.00	1.86	0.63	**6.37**

Accuracy (MCCM_easy_)		Pred freq.	True label							
71.78			C	B	E	G	I	H	S	T
True freq.		100%	21.14	1.39	21.25	3.69	0.04	30.86	9.81	11.81
Predicted label	C	13.51	**13.51**	0.21	0.54	0.03	0.46	0.32	0.54	5.52
	B	1.14	0.00	**0.05**	0.14	0.01	0.09	0.04	0.1	0.98
	E	18.58	0.00	0.15	**17.02**	0.01	0.35	0.17	0.33	3.22
	G	0.78	0.00	0.13	0.09	**0.68**	0.11	0.11	0.2	2.38
	I	2.12	0.00	0.00	0.00	0.00	**0.00**	0.00	0.00	0.03
	H	28.84	0.00	0.13	0.1	0.01	0.21	**27.74**	0.21	2.46
	S	4.12	0.00	0.24	0.47	0.02	0.6	0.28	**2.32**	5.88
	T	30.91	0.00	0.24	0.22	0.02	0.3	0.17	0.41	**10.45**

Accuracy (MCCM)		Pred freq.	True label							
83.74			C	B	E	G	I	H	S	T
True freq.		100%	21.14	1.39	21.25	3.69	0.04	30.86	9.81	11.81
Predicted label	C	23.31	**18.7**	0.02	0.45	0.31	0.00	0.38	0.56	0.71
	B	0.21	0.48	**0.10**	0.29	0.04	0.00	0.09	0.26	0.14
	E	22.1	0.48	0.03	**19.62**	0.11	0.00	0.1	0.63	0.30
	G	3.23	0.53	0.00	0.21	**1.73**	0.00	0.31	0.29	0.60
	I	0.00	0.02	0.00	0.00	0.01	**0.00**	0.00	0.00	0.01
	H	31.50	0.35	0.01	0.11	0.2	0.00	**29.72**	0.23	0.24
	S	7.76	1.8	0.05	0.96	0.33	0.00	0.48	**5.09**	1.10
	T	11.88	0.96	0.00	0.46	0.49	0.00	0.42	0.69	**8.79**

The bold values denote the best values of performance metrics.

The existing research results point out that, within different classes of all samples (either the classes is majority or minority), some examples are easier than others (Duggal et al., 2020). Comparing the performance of MCCM_easy_ and MCCM, we can find that the former based on loss function ${ℒ}_{\text{C}\_\text{easy}}$ can correctly classify the easier examples. Further, the latter, based on loss functions ${ℒ}_{\text{C}\_\text{easy}}$ and ${ℒ}_{\text{C}\_\text{hard}}$ , can correctly classify the easier examples and correctly classify the remaining harder examples. For example, from Table 3, we can find that MCCM_easy_ (optimized only by loss function ${ℒ}_{\text{C}\_\text{easy}}$ ) prediction accuracy of labels H, E, C, S, G, and B is much lower than that of MCCM (optimized by loss functions ${ℒ}_{\text{C}\_\text{easy}}$ and ${ℒ}_{\text{C}\_\text{hard}}$ ), which means the harder examples are further correctly classified. However, we also can find that the MCCM prediction accuracy of labels T and I is lower than that of MCCM_easy_, which may cause by the limited classifiers. On the whole, MCCM outperforms MCCM_easy_ by a large margin because MCCM is optimized by the loss functions ${ℒ}_{\text{C}\_\text{easy}}$ and ${ℒ}_{\text{C}\_\text{hard}}$ , increasing the loss value of the harder samples and inducing the model to pay more attention to them. Finally, the overall classification performance of the model is improved. Hence, the most harder samples (tend to be classified incorrectly by MCCM_easy_) are further classified correctly by MCCM, which can be known in the shown tables.

Associating the performance of MCCM_dir_ and MCCM, we can induce that if we can design a method to distinguish the samples well (the discrimination effect is close to using label information), our method can obtain the state-of-the-art performance. Future research can focus on this point.

## Conclusion

In the field of bioinformatics, understanding protein secondary structure is very important for exploring diseases treatments. This study proposes a framework for predicting the protein secondary structure, consisting of multilevel features extraction, multistage combination classifiers, and multilevel samples discriminating module. In the multilevel features extraction module, we design a different backbone network to extract the features of the multilevel (easy and hard levels in this study) from the original data. In the multistage combination classifiers module, we design two classifiers to deal with samples with different difficulty levels, respectively. Finally, in the multilevel samples discriminating module, we design a measurement standard based on the Dirichlet distribution and information entropy to assign suitable samples to different classifiers (multistage combination classifiers) with different levels. The first classifier is used to learn and classify the easier samples and filter them out, avoiding being sent to the second classifier. Further, the remaining harder samples will be sent to the second classifier. We compute the loss value of the two classifiers. Consequently, the loss value of the harder samples will be accumulated and will always be greater than the easier ones. This method can induce the model to pay more attention to harder samples and improve the classification performance. The experimental results on the publicly available benchmark CB513 dataset show the superior performance of the proposed method.

However, the experimental results show that the current multilevel samples discriminating the module in this study are not designed well, which limits the performance of our framework. Herein, the related experiments show that if the multilevel samples discriminating module is designed well, our framework can obtain state-of-the-art performance. Besides, the depth of our network and the number of classifier branches also can be further increased to raise the performance of our framework. Hence, future work can focus on designing a more effective multilevel samples discriminating module and designing the deeper network as well as the more classifier branches to further improve the model performance.

## Data Availability

The original contributions presented in the study are included in the article/Supplementary Material, further inquiries can be directed to the corresponding author.

## References

[B1] Altschul S. F. Madden T. L. Schäffer A. A. Zhang J. Zhang Z. Miller W. (1997). Gapped BLAST and PSI-BLAST: a New Generation of Protein Database Search Programs. Nucleic Acids Res. 25 (17), 3389–3402. 10.1093/nar/25.17.3389 9254694 PMC146917

[B2] Cao K. Wei C. Gaidon A. Arechiga N. Ma T. (2019). “Learning Imbal-Anced Datasets with Label Distribution-Aware Margin Loss,” in Advances in Neural Information Processing Systems, 1565–1576.

[B3] Chawla N. V. Bowyer K. W. Hall L. O. Kegelmeyer W. P. (2002). Smote: Synthetic Minority Over-sampling Technique. J. Artif. Intelligence Res. 16, 321–357. 10.1613/jair.953

[B4] Cuff J. A. Barton G. J. (2000). Application of Multiple Sequence Alignment Profiles to Improve Protein Secondary Structure Prediction. Proteins 40, 502–511. 10.1002/1097-0134(20000815)40:3<502:AID-PROT170>3.0.CO;2-Q 10861942

[B5] Cuff J. A. Barton G. J. (1999). Evaluation and Improvement of Multiple Sequence Methods for Protein Secondary Structure Prediction. Proteins 34, 508–519. 10.1002/(SICI)1097-0134(19990301)34:4<508:AID-PROT10>3.0.CO;2-4 10081963

[B6] Cui Y. Jia M. Lin T. Y. Song Y. Belongie S. (2019). “Class-balanced Loss Based on Effective Number of Samples,” in Proceedings of the IEEE Conference on Computer Vision and Pattern Recognition, 9268–9277. 10.1109/cvpr.2019.00949

[B7] Dempster A. P. (2008). “A Generalization of Bayesian Inference,” in Classic Works of the Dempster-Shafer Theory of Belief Functions (Berlin, Germany: Springer), 73–104. 10.1007/978-3-540-44792-4_4

[B8] Drori I. Dwivedi I. Shrestha P. Wan J. Wang Y. He Y. (2018). High Quality Prediction of Protein Q8 Secondary Structure by Diverse Neural Network Architectures. arXiv [Preprint]. arXiv:1811.07143.

[B9] Drozdetskiy A. Cole C. Procter J. Barton G. J. (2015). JPred4: a Protein Secondary Structure Prediction Server. Nucleic Acids Res. 43, W389–W394. 10.1093/nar/gkv332 25883141 PMC4489285

[B10] Duggal R. Freitas S. Dhamnani S. Chau D. H. Sun J. (2020). ELF: An Early-Exiting Framework for Long-Tailed Classification. arXiv [Preprint]. arXiv: 2006.11979.

[B11] Fang C. Li Z. Xu D. Shang Y. (2020). MUFold-SSW: a New Web Server for Predicting Protein Secondary Structures, Torsion Angles and Turns. Bioinformatics 36, 1293–1295. 10.1093/bioinformatics/btz712 31532508 PMC8489430

[B12] Fang C. Shang Y. Xu D. (2018). MUFOLD-SS: New Deep Inception-Inside-Inception Networks for Protein Secondary Structure Prediction. Proteins 86, 592–598. 10.1002/prot.25487 29492997 PMC6120586

[B13] Feng F. L. Chen H. M. He X. L. Ding J. Sun M. Chua T. S. (2019). Enhancing Stock Movement Prediction with Adversarial Training. California: IJCAI, 5843–5849.

[B14] Guo Z. Hou J. Cheng J. (2021). DNSS2 : Improved Ab Initio Protein Secondary Structure Prediction Using Advanced Deep Learning Architectures. Proteins 89, 207–217. 10.1002/prot.26007 32893403 PMC7790842

[B15] Hanson J. Paliwal K. Litfin T. Yang Y. Zhou Y. (2019). Improving Prediction of Protein Secondary Structure, Backbone Angles, Solvent Accessibility and Contact Numbers by Using Predicted Contact Maps and an Ensemble of Recurrent and Residual Convolutional Neural Networks. Bioinformatics 35, 2403–2410. 10.1093/bioinformatics/bty1006 30535134

[B16] Heffernan R. Yang Y. Paliwal K. Zhou Y. (2017). Capturing Non-local Interactions by Long Short-Term Memory Bidirectional Recurrent Neural Networks for Improving Prediction of Protein Secondary Structure, Backbone Angles, Contact Numbers and Solvent Accessibility. Bioinformatics 33, 2842–2849. 10.1093/bioinformatics/btx218 28430949

[B17] Huang Y. Chen W. Dotson D. L. Beckstein O. Shen J. (2016). Mechanism of Ph-dependent Activation of the Sodium-Proton Antiporter Nhaa. Nat. Commun. 7, 12940. 10.1038/ncomms12940 27708266 PMC5059715

[B18] Jens M. Michael M. Anita Z. Felix S. (2001). Generation and Evaluation of Dimension-Reduced Amino Acid Parameter Representations by Artificial Neural Networks. J. Mol. Model. 7 (9), 360–369.

[B19] Jones D. T. (1999). Protein Secondary Structure Prediction Based on Position-specific Scoring Matrices 1 1Edited by G. Von Heijne. J. Mol. Biol. 292 (2), 195–202. 10.1006/jmbi.1999.3091 10493868

[B20] Josang A. (2016). Subjective Logic: A Formalism for Reasoning under Uncertainty. Berlin, Germany: Springer. 978-3-319-42337-1.

[B21] Kabsch W. Sander C. (1983). Dictionary of Protein Secondary Structure: Pattern Recognition of Hydrogen-Bonded and Geometrical Features. Biopolymers 22, 2577–2637. 10.1002/bip.360221211 6667333

[B22] Källberg M. Wang H. Wang S. Peng J. Wang Z. Lu H. (2012). Template-based Protein Structure Modeling Using the Raptorx Web Server. Nat. Protoc. 7, 1511–1522. 10.1038/nprot.2012.085 22814390 PMC4730388

[B23] Li X. Zhong C.-Q. Wu R. Xu X. Yang Z.-H. Cai S. (2021). RIP1-dependent Linear and Nonlinear Recruitments of Caspase-8 and RIP3 Respectively to Necrosome Specify Distinct Cell Death Outcomes. Protein Cell 12, 858–876. 10.1007/s13238-020-00810-x 33389663 PMC8563874

[B24] Li Z. Yu Y. (2016). Protein Secondary Structure Prediction Using Cascaded Convolutional and Recurrent Neural Networks. arXiv [Preprint]. arXiv:1604.07176.

[B25] Lyu Z. Wang Z. Luo F. Shuai J. Huang Y. (2021). Protein Secondary Structure Prediction with a Reductive Deep Learning Method. Front. Bioeng. Biotechnol. 9, 687426. 10.3389/fbioe.2021.687426 34211967 PMC8240957

[B26] Minlong P. Zhang Q. Xing X. Y. Gui T. Huang X. J. Jiang Y. G. (2019). Trainable Undersampling for Class-Imbalance Learning. Proc. AAAI Conf. Artif. Intelligence 33, 4707–4714.

[B27] Myers J. K. Oas T. G. (2001). Preorganized Secondary Structure as an Important Determinant of Fast Protein Folding. Nat. Struct. Biol. 8, 552–558. 10.1038/88626 11373626

[B28] Pauling L. Corey R. B. Branson H. R. (1951). The Structure of Proteins: Two Hydrogen-Bonded Helical Configurations of the Polypeptide Chain. Proc. Natl. Acad. Sci. 37, 205–211. 10.1073/pnas.37.4.205 14816373 PMC1063337

[B29] Quan L. Lv Q. Zhang Y. (2016). STRUM: Structure-Based Prediction of Protein Stability Changes upon Single-point Mutation. Bioinformatics 32 (19), 2936–2946. 10.1093/bioinformatics/btw361 27318206 PMC5039926

[B30] Shapovalov M. Dunbrack R. L. Jr Vucetic S. (2020). Multifaceted Analysis of Training and Testing Convolutional Neural Networks for Protein Secondary Structure Prediction. PLOS ONE 15 (5), e0232528. 10.1371/journal.pone.0232528 32374785 PMC7202669

[B31] Uddin M. R. Mahbub S. Rahman M. S. Bayzid M. S. (2020). Saint: Self-Attention Augmented Inception-Inside-Inception Network Improves Protein Secondary Structure Prediction. Bioinformatics 36, 4599–4608. 10.1093/bioinformatics/btaa531 32437517

[B32] Wang G. Dunbrack R. L. Jr. (2003). Pisces: a Protein Sequence Culling Server. Bioinformatics 19, 1589–1591. 10.1093/bioinformatics/btg224 12912846

[B33] Wang S. Peng J. Ma J. Xu J. (2016). Protein Secondary Structure Prediction Using Deep Convolutional Neural fields. Sci. Rep. 6, 1–11. 10.1038/srep18962 26752681 PMC4707437

[B34] Zhang B. Li J. Lü Q. (2018). Prediction of 8-state Protein Secondary Structures by a Novel Deep Learning Architecture. BMC Bioinformatics 19, 293. 10.1186/s12859-018-2280-5 30075707 PMC6090794

[B35] Zhang Y. (2008). I-tasser Server for Protein 3D Structure Prediction. BMC Bioinformatics 9, 40. 10.1186/1471-2105-9-40 18215316 PMC2245901

[B36] Zhou J. Troyanskaya O. (2014). “Deep Supervised and Convolutionalgenerative Stochastic Network for Protein Secondary Structure Prediction,” in International Conference on Machine Learning (Beijing: PMLR), 745–753.

